# Role of Inflammatory Mediators in the Pathogenesis of Epilepsy

**DOI:** 10.1155/2014/901902

**Published:** 2014-08-13

**Authors:** Tadayuki Shimada, Takako Takemiya, Hiroko Sugiura, Kanato Yamagata

**Affiliations:** ^1^Neural Plasticity Project, Tokyo Metropolitan Institute of Medical Science, 2-1-6 Kamikitazawa, Setagaya-ku, Tokyo 156-8506, Japan; ^2^Medical Research Institute, Tokyo Women's Medical University, Shinjuku, Tokyo 162-8666, Japan

## Abstract

Epilepsy is one of the most common chronic brain disorders worldwide, affecting 1% of people across different ages and backgrounds. Epilepsy is defined as the sporadic occurrence of spontaneous recurrent seizures. Accumulating preclinical and clinical evidence suggest that there is a positive feedback cycle between epileptogenesis and brain inflammation. Epileptic seizures increase key inflammatory mediators, which in turn cause secondary damage to the brain and increase the likelihood of recurrent seizures. Cytokines and prostaglandins are well-known inflammatory mediators in the brain, and their biosynthesis is enhanced following seizures. Such inflammatory mediators could be therapeutic targets for the development of new antiepileptic drugs. In this review, we discuss the roles of inflammatory mediators in epileptogenesis.

## 1. Introduction

Epilepsy is a chronic neurological disorder characterized by recurrent seizures and is often accompanied by cognitive deficits and mood disorders [1–3]. Approximately 50 million people worldwide have epilepsy. Because seizures are the result of uncontrollable neural excitation in the brain, epilepsy has been considered to primarily be a neuronal disease. The targeting of neuronal ion channels and both gamma-aminobutyric acid (GABA) and glutamate receptors has been the primary approach to eliminate seizures. However, studies that have focused exclusively on neurons fail to address the questions that arise from more complex models of epileptogenesis. To date, studies using animal models and human patients with epilepsy have shown that the pathogenesis of epilepsy may be associated with both neuronal and nonneuronal components such as glial cells [4], brain vasculature [5], and leucocytes from the periphery [6].

The aberrant regulation of glial functions can elicit seizures and promote epileptogenesis [4]. Glial abnormalities, including chronically activated astrocytes and microglia, glial scars, and various gliomas, are likely to form epileptic foci in the brain [4]. The mechanisms through which glial cells can promote epileptogenesis can include increased neuronal excitability and inflammatory processes. The key roles of inflammatory processes in relation to epilepsy have been clarified over the last decade. Studies of the mechanisms of antiepileptic drugs (AEDs) have focused on ion channels, transporters, and excitatory/inhibitory neurotransmission [7]. However, the anti-inflammatory effects of AEDs have received recent attention due to their relevance to antiepileptic properties. For example, carbamazepine and levetiracetam can reduce the expression of inflammatory mediators in glial cell cultures [8, 9]. Levetiracetam can also normalize the resting membrane potential of astrocytes increased by inflammatory mediators [8]. One of the anticonvulsive effects of levetiracetam could be mediated through suppression of astroglial activation by inflammatory mediators [8].

In addition, dysfunction of the blood-brain barrier (BBB) may be responsible for abnormal neuronal firing. Disruption of the BBB causes the leakage of serum protein and leucocyte invasion into the brain. These exogenous inflammatory mediators have the potential to lower seizure thresholds [4, 5, 10, 11], which could alter channel sensitivity, neurotransmitter uptake or release, and glia-associated regulation of extracellular environments, such as potassium concentration [4, 5, 10, 11]. Accordingly, brain inflammation is one of the etiological factors that promote epileptogenesis and ictogenesis.

In this review, we discuss seizure-induced inflammatory mediators and the mechanisms through which these factors exacerbate epilepsy.

## 2. Inflammatory and Immune Responses in Epilepsy

Direct anti-inflammatory treatments have been reported to suppress some type of epileptic seizures that are resistant to conventional AEDs. For example, adrenocorticotropic hormone (ACTH) has been a first-line treatment for infantile spasms [13]. Anti-inflammatory effects of increased steroid hormone by ACTH treatment could play a crucial role in the suppression of refractory epilepsy in West syndrome [14]. In addition, intravenous immunoglobulin (IVIG) can suppress seizures in some types of intractable epilepsy, an effect that may be partially mediated through a reduction in cytokines and a suppression of astrocyte activation [15, 16]. These drugs are also able to confer protection against seizures in mice with some types of epilepsy that are resistant to conventional AEDs [17]. Combined with antiglial functions of conventional AEDs described above, anti-inflammatory medication could be a new promising treatment for refractory epilepsy.

Research with rodent models of epilepsy has uncovered roles for brain inflammation in epileptogenesis and ictogenesis. Pharmacological or electrical stimulation produces epileptic seizures accompanied by robust inflammatory responses in the brains of rodents [18–32]. The administration of proinflammatory or anti-inflammatory reagents has also been used to elucidate the effects of inflammatory mediators on seizure latency, frequency, duration, and severity [33, 34]. For instance, lipopolysaccharide, a provocative agent for inflammation, exacerbates seizure severity [33], whereas an inhibitory peptide against high-mobility group box-1 (HMGB1) inflammatory mediator decreased acute and chronic seizure recurrence [34]. Transgenic mouse models have also been used to evaluate the relationship between inflammatory mediators and seizure severity. Mice overexpressing cytokines, such as tumor necrosis factor-*α* (TNF-*α*) or interleukin-6 (IL-6), within astrocytes developed age-dependent neurological dysfunctions including a reduction in seizure threshold, spontaneous seizure frequency, and neuronal cell loss [35, 36]. These studies suggest the idea that brain inflammation could promote seizures and epileptogenesis.

**Figure 1:**
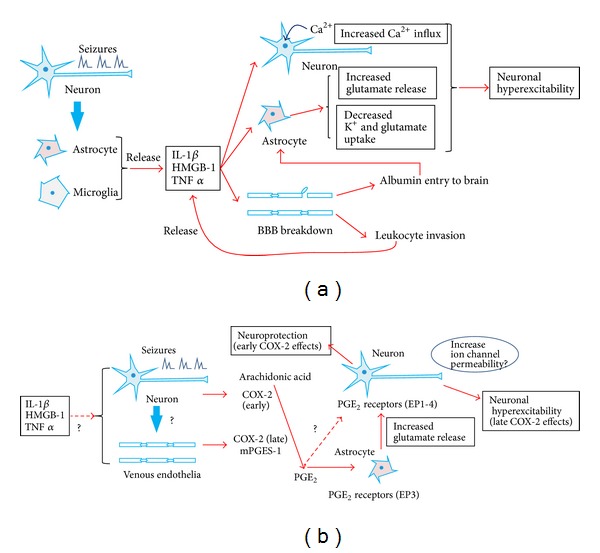
Proposed inflammatory mechanisms in epileptogenesis. (a) Epileptic seizures induce the release of cytokines from glial cells, thereby (1) increasing the influx of neuronal calcium; (2) enhancing extraneuronal glutamate concentration; (3) decreasing K^+^ and glutamate uptake by glia; and (4) impairing the BBB. BBB breakdown leads to albumin entry and leucocyte invasion into the brain, resulting in a further release of inflammatory cytokines. Such inflammatory responses cause an induction of neuronal hyperexcitability, reoccurrence of seizures, and finally the development of refractory epilepsy. (b) Seizures induce COX-2 in neurons (early phase) and vascular endothelial cells (late phase) and mPGES-1 in endothelial cells. These inducible PG synthases cooperate to produce PGE_2_, most likely in endothelial cells. Endothelial PGE_2_ might cause neuronal hyperexcitability by enhancing glutamate release from astrocytes via the glial EP3 receptor, whereas neuronal PGs may protect neurons against seizures.

Inflammatory cytokines, including interleukin-1*β* (IL-1*β*) and HMGB1, are released from astrocytes and microglia after seizures [22–24, 37]. IL-1*β* and HMGB1 activate IL-1R type I [38] and Toll-like receptor 4 (TLR4), respectively [39]. IL-1R/TLR signaling can regulate neuronal excitability, including the alteration of synaptic transmission, the reduction in GABA production, and the inhibition of outward current of Ca^2+^ channels [10, 40–42]. IL1R/TLR signaling may activate the Src kinase-mediated phosphorylation of* N*-methyl-D-aspartate (NMDA) receptor subunit 2B (NR2B) [43]. This phosphorylation can increase Ca^2+^ permeability of NMDA receptor [43]. Consequently, this NMDA-mediated Ca^2+^ influx could be enhanced in neurons, leading to increased neuronal excitability and excitotoxicity [34, 44]. HGMB1/TLR4 signaling also targets NR2B [36]. Thus, the activation of IL-1*β*/IL-1R or HMGB1/TLR4 signaling resulting from epileptic insults might result in a rapid change in neuronal excitability and a decreased seizure threshold (Figure 1(a)).

TNF-*α* is another inflammatory factor, and its expression is also upregulated following seizures [18, 36]. TNF-*α* is mainly released by microglia in the brain [45], and it can stimulate astrocytes to release glutamate [46]. An extracellular increase in glutamate concentration may stimulate glutamatergic neurons, thereby depolarizing their membrane potential. Taken together, postseizure production of inflammatory mediators can trigger neuronal hyperexcitability through modulations of ion channels and glutamate release in neurons and glia, respectively (Figure 1(a)).

In addition, the endothelial cells that make up the BBB could be another center for inflammatory processes in the brain. The activation of the IL-1*β* system following seizures induces neuronal cell loss and a breakdown in the BBB [47]. This breakdown leads to BBB leakage and the entry of albumin into the brain. Albumin entry can induce a further upregulation in inflammatory mediators and reduce the potassium and glutamate uptake of astrocytes, which leads to increased neural excitability [48–50]. BBB leakage also initiates the invasion of leucocytes and an enhancement in inflammatory reactions [51]. These findings indicate that a breakdown in the BBB can increase neuronal excitability by enhancing inflammatory responses in the brain (Figure 1(a)).

Taken together, epileptic seizures can provoke inflammatory responses, which enhance calcium influx into neurons, activate glial cells to increase extracellular potassium and glutamate, and induce further inflammatory response via BBB break down. These inflammatory responses may promote neural hyperexcitability and decrease the seizure threshold. Consequently, epileptic seizures and inflammatory mediators can form a positive feedback loop, reinforcing each other (Figure 1(a)).

## 3. Prostaglandin Production in Epilepsy

In addition to inflammatory cytokines, prostaglandins (PGs) are major factors that stimulate inflammation processes. PGs are known to markedly increase following seizures and may contribute to epileptogenesis and reduction in seizure threshold [10, 52]. Phospholipase A_2_ liberates arachidonic acid from membrane phospholipids. The enzyme cyclooxygenase (COX) converts arachidonic acid to PGH_2_, and the specific PG synthase converts PGH_2_ to various PGs such as thromboxane A_2_, PGF_2a_, PGE_2_, PGI_2_, or PGD_2_. Consistent with the robust production of PGs in the brain following seizures, an inducible type of COX (COX-2), but not the constitutively expressed COX-1, is rapidly induced in the brain following seizures [16, 53].

Because PGs play an important role in inflammatory responses, the functions of PGs in epileptogenesis have been studied for a considerable amount of time [52, 54]; however, data on the roles of COX-2 in epilepsy appear to be bidirectional. For example, following an injection of kainic acid (KA), postseizure treatment with the selective COX-2 inhibitor NS-398 prevented neuronal cell loss in the hippocampus [55]. However, the administration of another COX-2 inhibitor, nimesulide, prior to an injection of KA strongly aggravated KA-induced seizures and increased the mortality rate [56]. Moreover, pretreatment with NS-398 increased KA-induced TUNEL-positive neuronal death [57]. These conflicting results might be partly explained by possible dual roles for COX-2, which has been shown to play early neuroprotective and late neurotoxic roles following seizures [58]. Thus, the timing of COX-2 inhibitor administration may be crucial when treating epilepsy (Figure 1(b)).

The bifunctional aspects of COX-2 in epileptogenesis can also be explained by the diversity of PGs. For instance, PGD_2_ and PGF_2*α*_ can exhibit anticonvulsive functions. PGD_2_ synthase H-PGDS-knockout (KO) mice or PGD_2_ receptor DP1R-KO mice showed more severe seizures after pentylenetetrazol (PTZ) treatment than wild type (WT) mice, whereas deficiencies of the other synthase L-PGDS or receptor DP2R did not alter seizure severity [59]. Additionally, the intracisternal administration of PGF_2*α*_ after KA treatment reduced the seizure score and mortality [60]. By contrast, PGE_2_ mainly functions as a promoter of epileptogenesis and ictogenesis. The administration of PGE_2_ receptor (EP) antagonists reduced seizure severity and neuronal injury following pilocarpine- or PTZ-induced seizures [61–63]. Three PGE_2_ synthase (PGES) genes have been identified, including membrane-bound PGES (mPGES)-1, mPGES-2, and cytosolic PGES (cPGES). mPGES-1 is induced in the venous endothelia of the brain following an injection of KA [64], and its expression is upregulated in conditions of inflammation, pain, and fever [16, 65, 66]. mPGES-2 and cPGES show constitutive levels of expression, and their physiological functions remain unknown. The finding that the activity of mPGES-1 is tightly coupled to that of COX-2 [67] could support the importance of PGE_2_ function under epileptic conditions.

Several lines of evidence suggest that PGE_2_ could have an important role in epileptic neuronal cell death and the reduction in seizure threshold. mPGES-1-KO mice show little postictal production of PGE_2_, and the lack of increased PGE_2_ production resulted in the promotion of neuronal survival following KA injection [64, 68]. Furthermore, the intraventricular injection of PGE_2_ decreased the latency to methylmalonate- (MMA-) induced seizures and increased the amplitude of spikes measured by an electroencephalogram (EEG) in rats [69]. Thus, COX-2-coupled PGE_2_ production can promote seizures through mechanisms that drive epileptogenesis.

**Figure 2:**
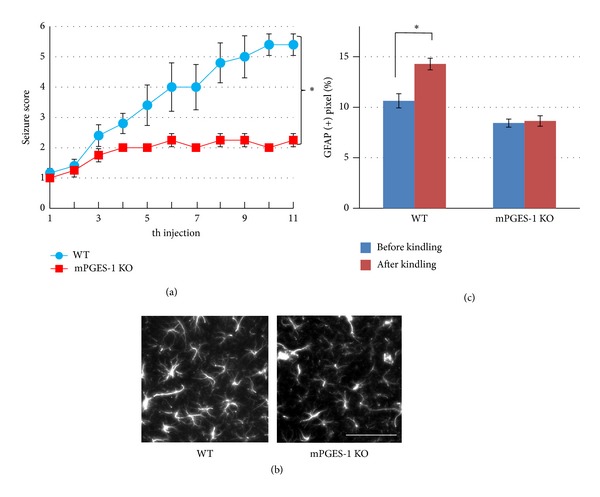
mPGES-1-KO mice show a less severe aggravation of epileptic seizures. (a) Development of epileptic convulsive seizures following consecutive treatments with PTZ. Two-month-old female C57/BL6 background mice of WT or mPGES-1-KO mice were injected intraperitoneally with a subconvulsive dose (35 mg/kg) of PTZ every other day. After each PTZ injection, the convulsive behaviors were observed for 30 min and the resultant seizures were classified and scored as follows: 0: normal behavior; 1: immobilization; 2: facial, forelimb, or hindlimb myoclonus; 3: continuous whole body myoclonus; 4: rearing, tonic seizure; 5: tonic-chronic seizure; and 6: death [12]. mPGES-1-KO mice showed significant reduction in seizure score compared with that in WT mice (∗: *P* < 0.05, repeated-measured ANOVA). (b) Representative images of the CA3 region of hippocampal sections from WT and mPGES-1-KO mice after PTZ-induced chemical kindling. Mice were fixed with 4% paraformaldehyde. Serial sections (30 *μ*m) were generated, and immunostaining was performed with a rabbit anti-GFAP antibody (DAKO). Gliosis was observed only in the sections from wild type mice. Scale bar = 20 *μ*m. (c) Quantification of gliosis development: 200 *μ*m × 200 *μ*m squares were randomly overlaid on the CA3 region of hippocampal slices and the percentage of pixels that had a higher intensity of GFAP signal than the threshold was compared. The GFAP-positive area was significantly increased after kindling in WT mice, whereas no increase was observed in mPGES-1-KO mice (*P < 0.01, two-way ANOVA followed by Tukey's test). Six squares were taken from one brain slice. Four slices were examined under each condition.

To determine whether mPGES-1-derived PGE_2_ is directly involved in epileptogenesis, we used mPGES-1-KO mice. To mimic the gradual exacerbation of epileptic seizures, a subconvulsive dose of PTZ was administered to WT or mPGES-1-KO mice every other day (PTZ chemical kindling). WT mice showed rapid increases in seizure severity, whereas mPGES-1-KO mice showed a significantly slower elevation in seizure score (Figure 2(a)). Immunohistochemical analyses revealed that glial fibrillary acidic protein- (GFAP-) positive astrocytes were increased in the hippocampal CA3 region of the wild type mice after the kindling, indicating that PGE_2_ production promotes reactive astrogliosis (Figure 2(b)). Interestingly, mPGES-1-KO mice did not show any increase in GFAP-positive astrocytes after the kindling (Figure 2(c)). Reactive astrogliosis is one of the pathological hallmarks of temporal lobe epilepsy, which include segmental neuronal loss and increases in GFAP-positive cells in the hippocampus and associated temporal lobe structures [4]. Our results indicate that mPGES-1-KO mice are resistant to the development of PTZ kindling and also to kindling-induced astrogliosis.

How can PGE_2_ regulate hyperexcitability and epileptogenesis? PGE_2_ acts on four G protein-coupled receptors named EP1, EP2, EP3, and EP4. EP receptors have been shown to be localized in the brain cells [70, 71]; however, it still remains unclear on which cells PGE_2_ acts to produce its epileptogenic effects. By contrast, pharmacological approaches have preceded the clarification of brain PGE_2_ function. The administration of a selective EP2 antagonist prevented neuronal cell loss after pilocarpine-induced seizures [62]. Furthermore, the pharmacological inhibition of other receptors, EP1, EP3, or EP4, resulted in increases in PTZ-induced seizure latency [63]. These results suggest that PGE_2_ may cause neuronal cell death and neuronal hyperexcitability via hippocampal EP receptors (Figure 1(b)).

The effects of PGE_2_ on neurons might be partly similar to those of other inflammatory mediators in terms of their enhancement of neuronal excitability. Several studies have shown that PG synthesis is upregulated by IL-1*β* treatment. In a lung fibroblast cell line, IL-1*β* induced mPGES-1 mRNA expression [72]. Additionally, IL-1*β* treatment increased COX-2 mRNA levels in cultured murine primary astrocytes [73]. Thus, inflammatory cytokines may promote PGE_2_ synthesis in the brain following epileptic seizures.

PGE_2_ can act on neuronal EP receptors directly. The application of PGE_2_ led to significant increases in firing frequency and excitatory postsynaptic potential (EPSP) amplitude in rat hippocampal slice cultures [74]. PGE_2_-induced increases in neuronal excitability may be partly attributable to an inhibition of potassium currents, resulting in boosted Ca^2+^ influx [74]. However, given that the mPGES-1 enzyme is preferentially expressed in the brain microvasculature [65], it may be more likely that PGE_2_ acts on glial cells but not on neurons. PGE_2_ appears to act on glial processes surrounding brain microvessels. We previously reported that the EP3 receptor was localized to astroglial end feet around microvessels [64]. Additionally, mPGES-1-KO mice showed decreased astrogliosis compared to WT mice following KA treatment [64]. These findings suggest that endothelial PGE_2_ may act on glial EP3 receptors and alter glial function. In fact, glutamate release from astrocytes was drastically reduced in mPGES-1-KO mice compared to WT mice [66, 75]. Thus, PGE_2_ is both an inflammatory mediator and a promoter of extracellular gliotransmission via astrocytic EP receptors [46] (Figure 1(b)).

In conclusion, epileptic seizures rapidly induce COX-2 mRNA in neurons and afterwards upregulate both COX-2 and mPGES-1 mRNAs in venous endothelia [16, 64–66]. Treatment with NS-398 and nimesulide prior to seizure induction aggravates neuronal damage in the hippocampus, whereas postseizure treatments with NS-398 prevent the damages [55, 56]. Neuronal COX-2-derived PGs appear to protect neurons against seizures, whereas endothelial PG (PGE_2_) might participate in seizure-induced neuronal cell death by stimulating astrocytes to release glutamate [58]. Thus, PG-mediated inflammation may also form a positive feedback loop to exacerbate epileptic seizures in a late phase after seizures (Figure 1(b)). Epileptic seizures rapidly induce COX-2 in excitatory neurons and increase brain PGE_2_ levels [64]. However, this neuronal upregulation of COX-2 and PGE_2_ is transient and simultaneous robust increases in brain PGD_2_ levels could surpass the effects of PGE_2_, resulting in suppression of seizures. By contrast, late induction of endothelial COX-2 and mPGES-1 lasts longer than early phase and this sustained production of PGE_2_ may promote neurodegeneration via astrocytes. Thus, time course and cell type-specific expression of COX-2 could cause the dual effects of PG production on epileptogenesis.

## 4. Conclusion and Perspective

Most current AEDs act on ion channels that directly control neuronal excitability. These medications have at least two major problems. First, even with optimal AED therapy, 20~30% of patients have poor seizure control and become intractable. Second, as these medications act as general central nervous system (CNS) depressants and must be taken chronically for seizure suppression, they also have marked inhibitory effects on cognitive development. Therefore, the development of new AEDs that can modulate seizures through another mechanism is required for refractory epilepsy treatment.

Animal models and clinical evidence have emphasized the involvement of inflammatory mediators in seizure susceptibility and epileptogenesis [76–78]. Robust and general inflammatory responses in the brain lower the seizure threshold, enhance neuronal excitability, increase BBB permeability, and promote epileptogenesis. COX-2 and mPGES-1 are known to play key roles in the inflammatory responses to insults and consequently increase postseizure inflammation and the resulting hyperexcitability of brain neurons. COX-2 inhibitors such as celecoxib have been developed and prescribed for chronic inflammatory pain and rheumatoid arthritis. Hence, they can be used in conjunction with AEDs to treat brain inflammation and reduce neuronal hyperexcitability. Although celecoxib suppresses epileptogenesis, for example, the development of PTZ-kindling, this inhibitor may be more effective for postseizure inflammation than for the prevention of seizure incidence. However, given that chronic celecoxib treatment can be accompanied by serious cardiovascular side effects [79], persistent COX-2 inhibition would not be beneficial as a therapeutic strategy for intractable epilepsy.

By contrast, the regulation of specific PG signaling should offer more selective actions and fewer complicating adverse effects. Although PGE_2_ synthase inhibitors may be good candidates for epilepsy treatment, specific inhibitors are still under development. Recently, attention has been paid to EP receptors. An EP2 inhibitor appears to suppress neurodegenerative pathology following epileptic seizures, and the inhibition of the other EP receptors, EP1, EP3, and EP4, might delay seizure induction [61–63]. The discovery of selective EP receptor antagonist(s) could contribute to avoid the serious side effects of chronic COX-2 inhibition and provide more selective therapies for refractory epilepsy.

Modulators of PG receptor function could also improve our understanding of the roles of PGs in the brain immune system and inflammation. Consequently, more precise molecular roles of PGs in epileptogenesis will need to be clarified. This knowledge will help to develop newer and better treatments and medications for epilepsy.
